# Exploring the Role of ChatGPT-4, BingAI, and Gemini as Virtual Consultants to Educate Families about Retinopathy of Prematurity

**DOI:** 10.3390/children11060750

**Published:** 2024-06-20

**Authors:** Ceren Durmaz Engin, Ezgi Karatas, Taylan Ozturk

**Affiliations:** 1 Department of Ophthalmology, Izmir Democracy University, Buca Seyfi Demirsoy Education and Research Hospital, Izmir 35390, Turkey; 2 Department of Biomedical Technologies, Faculty of Engineering, Dokuz Eylul University, Izmir 35390, Turkey; 3 Department of Ophthalmology, Agri Ibrahim Cecen University, Agri 04200, Turkey; e.karatas.2015@gmail.com; 4 Department of Ophthalmology, Izmir Tinaztepe University, Izmir 35400, Turkey; ataylan6@yahoo.com

**Keywords:** artificial intelligence, BingAI, ChatGPT, Gemini, large language models, retinopathy of prematurity

## Abstract

Background: Large language models (LLMs) are becoming increasingly important as they are being used more frequently for providing medical information. Our aim is to evaluate the effectiveness of electronic artificial intelligence (AI) large language models (LLMs), such as ChatGPT-4, BingAI, and Gemini in responding to patient inquiries about retinopathy of prematurity (ROP). Methods: The answers of LLMs for fifty real-life patient inquiries were assessed using a 5-point Likert scale by three ophthalmologists. The models’ responses were also evaluated for reliability with the DISCERN instrument and the EQIP framework, and for readability using the Flesch Reading Ease (FRE), Flesch-Kincaid Grade Level (FKGL), and Coleman-Liau Index. Results: ChatGPT-4 outperformed BingAI and Gemini, scoring the highest with 5 points in 90% (45 out of 50) and achieving ratings of “agreed” or “strongly agreed” in 98% (49 out of 50) of responses. It led in accuracy and reliability with DISCERN and EQIP scores of 63 and 72.2, respectively. BingAI followed with scores of 53 and 61.1, while Gemini was noted for the best readability (FRE score of 39.1) but lower reliability scores. Statistically significant performance differences were observed particularly in the screening, diagnosis, and treatment categories. Conclusion: ChatGPT-4 excelled in providing detailed and reliable responses to ROP-related queries, although its texts were more complex. All models delivered generally accurate information as per DISCERN and EQIP assessments.

## 1. Introduction

Retinopathy of prematurity (ROP) predominantly affects premature infants, leading to significant visual disabilities or even blindness [1]. Awareness and understanding of ROP are essential not only for health professionals but also for parents of premature infants [2]. Despite parents playing a vital role in managing ROP, most of the screening, diagnostic, and treatment procedures are conducted in their absence. This situation, combined with the inherent risks associated with ROP, can cause significant parental anxiety and drive a need for reliable information [3]. In busy clinical settings, healthcare providers often struggle to comprehensively respond to all questions from parents. Consequently, many parents resort to seeking information from alternative, readily accessible sources [4]. Seeking health information online has become a popular method due to its broad availability, affordability, convenience, anonymity, and interactivity. Recent statistics indicate that in European Union countries, the prevalence of online health seeking has increased from 33% to 56% in the last 10 years [5]. This behavior can significantly influence the dynamics of patient–doctor interactions, the utilization of healthcare services, and decision-making process. Moreover, when the health concern involves a child, parents may be particularly susceptible to the effects of misinformation or a lack of information. A systematic review of online health information seeking by parents for their children reported high levels of parental health anxiety, with prevalence rates ranging from 14% to 52% [6]. Therefore, ensuring that parents have access to accurate and reliable information is crucial.

Artificial intelligence (AI) chatbots are software applications crafted to mimic human dialogue by employing natural language processing and machine learning to process and respond to various inquiries [7]. In recent years, they have become increasingly useful in several domains, including education, customer service, entertainment, finance, and medicine. Even in the field of ophthalmology, several studies have evaluated the accuracy of LLMs in answering patient inquiries and providing scientific medical information to healthcare workers about various ophthalmic conditions, such as myopia [8], glaucoma [9], amblyopia [10], retinal diseases [11], and cataracts [12]. Given the pervasive use of smartphones and the internet, chatbots are not only affordable and accessible but also continuously evolving based on the feedback they obtain. Consequently, the deployment of chatbots, both commercially and for personal use, is anticipated to grow substantially in the foreseeable future [7]. ChatGPT-4, BingAI, and Gemini are at the forefront of AI-driven conversational agents, each engineered by its developers—OpenAI, Microsoft, and Google—to fulfill specific roles and seamlessly integrate into various digital environments.

ChatGPT-4 (www.chatgpt.com, accessed on 19 April 2024), developed by OpenAI, is an advanced model in the Generative Pre-trained Transformer series, utilizing vast datasets and fine-tuning through supervised and reinforcement learning from human feedback [13]. It excels at creating human-like text and handling complex tasks across various topics. BingAI (www.bing.com, accessed on 19 April 2024), another LLM using a variant of the GPT model adapted for research-centered capabilities, is integrated with Microsoft’s Bing search engine to enhance real-time search responses, aiming to transform search experiences with direct answers and content summaries [14]. On the other hand, Google’s Gemini (gemini.google.com, accessed on 19 April 2024), built on the Language Model for Dialogue Applications (LaMDA), produces informative and conversational content continuously updated with the latest web information [15]. Each of these models—ChatGPT-4 with its broad conversational capabilities, BingAI with its research-centric prowess, and Gemini with its real-time information synthesis—reflects the strategic priorities of their respective developers and offers distinct advantages depending on the application. Therefore, each may behave differently in response to patient inquiries about medical conditions [16,17]. Similar studies in the ophthalmology literature also report varying results regarding the success of these LLMs in providing professional medical information or responding to patient inquiries [8,9,10,11,12]. Moreover, there is a lack of information about the accuracy and readability of LLMs in addressing ROP-related patient questions. Therefore, the aim of this study is to evaluate the effectiveness of three large language models (LLM), ChatGPT-4, BingAI, and Gemini, in providing accurate, reliable, and readable responses to patient inquiries about ROP.

## 2. Materials and Methods

### 2.1. The Inquiries and Responses

A collection of 50 real-life patient questions sourced from parents during ROP screening appointments was assembled. The questions were organized into five key sections: general information, screening, diagnosis, treatment, and prognosis of ROP, with each section composed of 10 questions. On 10 April 2024, the three LLMs, ChatGPT-4, BingAI, and Gemini, were instructed with the following prompt: “Assume you’re an experienced ophthalmologist specialized in ROP, and I am the parent of a premature baby who will undergo a ROP examination. Can you please answer my questions?”. Then, after being rigorously checked for grammatical accuracy, the questions were presented to the LLMs one by one. Due to the LLMs’ inherent ability to produce varied responses to the same query, only the first response to each question was recorded for analysis. The responses were unrestricted by the word count to allow for thorough explanations and did not require citation of reference articles, as they were intended for a non-medical audience, namely parents of patients screened for ROP. This study was approved by the local ethics committee.

### 2.2. The Assessment Scales

The accuracy of the responses was assessed by comparing them to current healthcare guidelines for ROP. This evaluation was conducted by a panel of three ophthalmologists (C.D.E., E.K., and T.O.), who possess experience in ROP ranging from 5 to 15 years and are currently in charge of the ROP Units at their respective hospitals. The panel of three ophthalmologists was blinded to which LLM’s responses were being evaluated. A 5-point Likert scale, previously used in evaluating the accuracy of LLMs, was employed for this purpose [17,18]. Discrepancies in Likert scale ratings were resolved through discussion based on guidelines, with the consensus score accepted as the final rating by the ophthalmologists. The scoring proceeded as follows:

1. Strongly disagreed: Very poor or unacceptable inaccuracies and high risk of harm.
2. Disagreed: Poor accuracy with potentially harmful mistakes.
3. Neither agreed nor disagreed: Moderate inaccuracies that could be misinterpreted, presenting negligible harm.
4. Agreed: Good quality responses with only minor, nonharmful inaccuracies.
5. Strongly agreed: Very good accuracy, devoid of any inaccuracies, no risk of harm.

The responses were further assessed using the established DISCERN and EQIP scales to evaluate their accuracy and reliability. The DISCERN instrument, developed by Charnock et al. [19], is designed to assess the quality of health information resources, with a particular focus on their reliability and the content’s relevance to treatment options. It consists of 16 questions, each rated on a scale from 1 to 5, where 1 indicates significant shortcomings and 5 indicates minimal shortcomings. The first part of the tool evaluates the resource’s reliability through eight questions; the second part assesses the specifics of the treatment for the disease through seven questions, and the final question evaluates the overall quality. The total score, excluding the last question, ranges from 16 to 75 and is used to categorize the quality into five levels: excellent (63–75), good (51–62), moderate (39–50), poor (27–38), or very poor (16–26). The EQIP tool, developed by health professionals and patient information managers, provides a comprehensive framework for assessing the quality of health information resources, such as websites and patient leaflets [20]. It features 20 items that examine various aspects, including accuracy, balance, structure, and design. Each item is evaluated with a simple yes-or-no decision, leading to a maximum possible score of 100. Although the category to be deducted according to the score is not specified exactly, such as DISCERN, the scores found in various studies are described as fair, moderate, or excellent [21,22].

Finally, the comprehensibility and complexity of the responses were evaluated using three widely recognized readability scales: the FRE score, the FKGL, and the Coleman–Liau Index [23]. The FRE score, which ranges from 0 to 100, measures text comprehensibility, with higher scores indicating greater readability. Conversely, the FKGL and the Coleman–Liau Index assess the educational level required to understand the text and its complexity, respectively, where higher scores denote more complex content.

In the evaluations conducted with the DISCERN, EQIP, and the three readability scales, all responses from each LLM were collectively analyzed to derive a single composite score for each model.

### 2.3. Statistical Analysis

Statistical analyses were performed using SPSS Statistics Version 25 (IBM, Armonk, NY, USA). Descriptive statistics were employed to summarize the dataset, with categorical variables expressed as counts and percentages, and quantitative variables presented as means ± standard deviations or medians (minimum–maximum). The non-parametric Friedman test with Bonferroni correction was utilized for comparisons among the LLMs. If a significant difference was found among the three LLMs, pairwise comparisons were conducted using the Wilcoxon rank-sum test. A *p*-value of less than 0.05 was considered statistically significant.

## 3. Results

In assessing responses to a set of 50 questions, divided into 5 subcategories with 10 questions each, it was found that no LLM scored 1 point on any question using the Likert scale. ChatGPT-4 achieved the highest rating of 5 points in 45 questions (90%), significantly outperforming BingAI and Gemini, which scored similarly in 15 (30%) and 19 (38%) of the questions, respectively. ChatGPT-4 received a score of 2 points (disagreed) for only one question, whereas BingAI and Gemini received this score for 6 and 9 questions, respectively. Moreover, considering responses categorized as “agreed” (4 points) or “strongly agreed” (5 points), 49 responses (98%) from ChatGPT-4 fell into this range, compared to 39 responses (78%) from BingAI and 35 responses (70%) from Gemini. The distribution of Likert scores for the three LLMs in providing satisfactory answers across various question subcategories is presented in Table 1.

Upon reviewing the responses across all questions, irrespective of subcategory, significant variances in Likert scores among the three models were noted, primarily due to ChatGPT-4’s higher median score. In the domain of general knowledge about ROP, no significant differences were detected between the models (*p* = 0.735). However, significant differences were noted between ChatGPT-4 and BingAI (*p* = 0.005) as well as between ChatGPT-4 and Gemini (*p* = 0.010) in the screening subcategory scores. Similar significant discrepancies were observed between ChatGPT-4 and BingAI (*p* = 0.024) as well as between ChatGPT-4 and Gemini (*p* = 0.011) in the diagnosis subcategory scores, and between ChatGPT-4 and BingAI (*p* = 0.007) as well as between ChatGPT-4 and Gemini (*p* = 0.010) in the treatment subcategory scores. No significant differences were found between BingAI and Gemini in the screening (*p* = 0.107), diagnosis (*p* = 1.000), and treatment (*p* = 0.131) subcategory scores. On the other hand, there were significant differences in the prognosis subcategory scores between ChatGPT-4 and BingAI (*p* = 0.010) and between Gemini and BingAI (*p* = 0.035), but no significant difference was observed between ChatGPT-4 and Gemini (*p* = 0.590). ChatGPT-4 achieved its highest median scores in the “diagnosis” and “prognosis” subcategories, whereas BingAI and Gemini scored highest in the “general information” subcategory. Table 2 displays the mean and median scores of the three LLMs across various question categories.

In terms of reliability, ChatGPT stands out among the three evaluated large language models, achieving the highest scores on both the DISCERN (63 points) and EQIP (72.2 points, rated as excellent) scales. These scores indicate that it provides the most dependable and highest-quality health information. Conversely, BingAI records the lowest scores on these reliability assessments, with 53 points in DISCERN (rated as good) and 61.1 (rated as moderate) in EQIP, suggesting a relative shortfall in the quality and accuracy of the health information it provides, though these scores are still considered more than fair. Regarding readability, Gemini is distinguished by having the highest FRE score at 39.1, indicating it is the easiest to read among the three models. However, ChatGPT exhibits the most complex text structure, reflected by the highest scores on both the Coleman-Liau Index of 15.27 and the FKGL of 13.5, suggesting that its outputs are suited for a more advanced reading level. The reliability and readability of the responses provided by the three LLMs are summarized in Table 3.

## 4. Discussion

Large language models have ascended to prominence in the medical area owing to their proficient, rapid information retrieval and algorithmic decision-making capabilities. Not only are they a source of information for patients, but they also help the medical staff to answer electronic patient messages as they are easy to use, fast and reliable with needed modifications [24]. One study found that individuals were overall willing to receive health advice from an LLM, especially for low-risk topics, which means that LLMs could be seen as an alternative source of information when an actual health care professional is not available [25]. Because ROP is a serious disease that can potentially lead to blindness, using LLMs as sources of information can significantly increase family anxiety over even the slightest incorrect answer. To the best of the authors’ knowledge, this is the first cross-sectional study to assess the accuracy and readability of responses provided by LLMs to common patient questions about ROP.

This study found that ChatGPT provided more detailed and accurate responses to patient questions about ROP, with 98% of answers falling into the “agreed” or “strongly agreed” category compared to BingAI and Gemini. A similar result was found by Coskun et al. [16] in questions about methotrexate use, as ChatGPT achieved a 100% correct answer rate, while Bard (currently known as Gemini) and BingAI scored 73.91%. In another study assessing the quality and readability of AI chatbot-generated answers to frequently asked clinical inquiries in the field of bariatric and metabolic surgery, a significant difference was observed in the proportion of appropriate answers among the three LLMs: ChatGPT-4 led with 85.7%, followed by Bard at 74.3%, and BingAI at 25.7% [26]. Those results may stem from the key differences in design and objectives of the three LLMs. ChatGPT-4 is trained on a diverse dataset with a focus on creating human-like conversational experiences, employing both supervised and reinforcement learning to produce contextually rich responses. In contrast, BingAI and Gemini are optimized for search and information retrieval, emphasizing brevity and directness. This emphasis on quick fact retrieval leads to less detailed responses and a lack of important information. In contrast, ChatGPT-4 employs a narrative-driven content approach designed for in-depth educational interactions, providing more exhaustive and informative responses. Additionally, relying on web searches, BingAI and Gemini may source information from various websites, some of which lack scientific credibility, unlike ChatGPT, which utilizes its own extensive database that also includes scientific articles up to a certain date. This might account for the higher number of disagreed responses from BingAI (in 6 questions) and Gemini (in 9 questions) compared to only 1 from ChatGPT-4. Conversely, there are several studies in which BingAI or Gemini either outperformed ChatGPT-4 or where no significant differences were observed among the three LLMs in terms of patient inquiries [27,28]. These variations could be attributed to the nature of the questions, the expected depth of the answers, or the different scoring systems used in these studies.

There were no differences between the median scores of LLMs in the “general information” subcategory, which focused on definitions and prevalences. This lack of variance can likely be attributed to the standardized and well-known nature of disease definitions, which are straightforward in the LLMs’ training data, resulting in similar outputs across all models [29]. In contrast, the subcategories of screening, diagnosis, treatment, and prognosis exhibited variability in median scores among the three LLMs. This discrepancy can be explained by the likelihood of receiving inputs with conflicting information, as well as the updating capabilities of LLMs, as diagnostic criteria and treatment methods are continuously evolving and the prognosis often varies with the introduction of new treatments [30,31].

The highest DISCERN and EQIP scores were observed in responses generated by ChatGPT-4, in line with its top Likert scores among the three LLMs evaluated. In a study on ChatGPT-4’s efficacy in providing information about periodontal diseases to patients, the responses were rated as ‘good’ based on total DISCERN scores [32]. Similarly, in a study assessing the quality of AI-generated medical information on appendicitis, ChatGPT-4 and Bard received DISCERN scores of 62.0 and 62.3, respectively, categorized as having “good” accuracy [33]. Our findings are consistent with these studies, as both BingAI and Gemini were rated as “good” while ChatGPT-4’s responses achieved “excellent” accuracy according to the DISCERN scale. In another study, the EQIP scores for ChatGPT’s responses to common questions about osteoporosis varied significantly, ranging from 36.36 to 61.76, with a mean score of 48.71, indicating “serious problems with quality” [34]. In contrast, the EQIP scores in our study ranged from 61.1 to 72.2, demonstrating very good accuracy.

In terms of readability, Gemini’s responses were the easiest to read on average, though it was still classified as “difficult” with FRE score of 39.1. In contrast, both ChatGPT-4 and BingAI produced answers that were categorized as “extremely difficult” and corresponded to a college-graduate reading level according to the FRE. Similarly, a study investigating the role of LLMs in patient inquiries about bariatric surgery found BingAI to be the hardest and Gemini the easiest to read, echoing our findings [26]. Another study assessing the readability of three LLMs reported a FKGL score of 9.7 for Bard and 10.15 for ChatGPT, suggesting Bard requires a “professional level” of understanding and ChatGPT is “very difficult to read”. However, BingAI received the highest FKGL score, indicating that it was the most complex to understand [17]. Similarly, we found BingAI to be the most complex to comprehend, possibly due to its academic research-centered design [14]. Moreover, the variations in readability scores among Gemini, ChatGPT-4, and BingAI can largely be attributed to their specific design goals and the nature of their training data. Gemini is tailored for conversational clarity, resulting in content that is easier to read, as indicated by higher FRE scores. On the other hand, ChatGPT-4 and BingAI, which are trained on more diverse and complex datasets, prioritize detailed accuracy and depth. This approach results in outputs that are denser and more challenging to read, aligning with a college-graduate level per the FRE scale [14,15]. In our opinion, readability is as important as the accuracy of information provided by LLMs. Families seeking medical advice from artificial intelligence would be disadvantaged by an LLM that provides responses at an advanced reading level, rendering the information incomprehensible to the general population. Therefore, it is imperative that developers of LLMs prioritize not only the accuracy and accessibility of the information provided but also its understandability for a broader audience.

Limitations of this study include the following: the questions were posed only once without rephrasing or requesting clarifications, and the LLMs were not permitted to correct themselves. Also, this study represents a single time-point analysis, implying that the accuracy and readability of the responses could vary over time. As LLMs evolve, it is crucial to continuously evaluate their accuracy, their ability to avoid spreading misinformation, and their capacity to learn from feedback to maintain the highest standards of safety and reliability. Another potential limitation is the subjectivity inherent in the evaluation process since the other physicians might have provided different assessments. To mitigate this risk, we employed a consensus method involving qualified experts in ROP. Moreover, despite ChatGPT-4’s strong overall performance, its inability to accurately define ROP highlights a notable deficiency. The response to this fundamental question should have been given greater weight in the evaluation process. However, all questions were given the same weight due to the nature of the Likert system used in this study, which may present a limitation. Nevertheless, the primary strengths of this study are its novelty and rigorous methodology—it is the first to evaluate the accuracy and intelligibility of responses from the three most commonly used LLMs to patient inquiries about ROP. We utilized established, validated scales for assessment and drew upon the expertise of three experienced ROP specialists who scored the answers with consensus. Additionally, the high number of patient questions evaluating distinct aspects of ROP is another strength of our study.

In the swiftly evolving field of LLMs, we are observing a trend towards the integration of broad, general-purpose models with those specialized for specific domains. The recent introduction of Med-PaLM suggests an impending era where AI tools are finely crafted for healthcare [35]. Similarly, the development of GastroGPT, a domain-specific LLM focused on gastroenterology, is underway [36]. In the foreseeable future, a LLM centered around ophthalmology literature could enhance both the precision and reliability of information for ophthalmologists and patients dealing with eye conditions. Additionally, as the ability of LLMs to simplify information improves, making it accessible even to patients with the lowest literacy levels, the understandability of the responses will increase.

## 5. Conclusions

The findings of our study reveal that AI-driven LLMs, notably those using the GPT-4 architecture such as ChatGPT-4, demonstrate significant potential as dependable tools for accurately addressing questions about ROP. Although these models can be complex to read, their accuracy and reliability are noteworthy. While they are not intended to replace human expertise, these AI models have the potential to be integrated into clinical practice to enhance the efficiency and availability of information for both healthcare professionals and patients. This integration could facilitate better-informed decision-making and improve patient outcomes by providing timely and accurate responses to medical inquiries. However, future research, encompassing a broader range of patient queries across all domains of ROP, and involving blinded participants, is necessary to validate these findings and ensure the effectiveness and reliability of AI models in clinical settings.

## Figures and Tables

**Table 1:** Scores of responses provided by large language models to each question, evaluated on a Likert scale.

	ChatGPT-4	BingAI	Gemini
**General Information about Retinopathy of Prematurity (ROP)**			
What exactly is ROP?	2	5	5
2. How common is retinopathy of prematurity among babies?	5	2	5
3. What are the chances my premature baby will develop ROP?	5	5	5
4. Are there specific factors that increase the risk of my baby developing ROP?	5	5	5
5. Is there a genetic component to ROP, or is it strictly related to prematurity and other risk factors?	5	5	5
6. What are the first signs of ROP I should look out for?	5	5	4
7. What does mild and severe ROP mean?	5	4	3
8. Can you explain what “plus disease” in ROP means?	5	5	5
9. What is aggressive ROP and how is it different from other forms?	5	5	5
10. Is ROP a blinding disease?	5	5	5
**Screening of Retinopathy of Prematurity**			
How is the ROP screening performed?	5	4	2
2. How frequently does my baby need to be checked for ROP?	5	4	2
3. Until when should my baby continue to have ROP screenings?	4	3	2
4. Will the examination for ROP be painful for my baby?	5	2	2
5. Are there any other physiological changes except for pain in my baby during an ROP exam?	5	2	2
6. Am I allowed to be with my baby during the ROP screening?	5	4	3
7. Are there any side effects from the ROP screening tests?	4	4	3
8. How soon do the side effects from screening go away, and will my baby need treatment for them?	4	3	4
9. Why does my baby, who was born full-term, need an ROP exam after a NICU stay?	5	4	3
10. What should I bring or prepare for my child’s ROP screening appointment?	5	4	5
**Diagnosis of Retinopathy of Prematurity**			
What happens if ROP is detected in my baby?	5	5	4
2. If one of my baby’s eyes is diagnosed with ROP, what’s the chance the other eye is also affected?	5	5	5
3. Besides the regular examination conducted by doctors, are there any additional diagnostic tests used to confirm ROP in infants?	5	2	4
4. How do doctors decide how severe the ROP is and whether it’s mild or severe?	5	4	2
5. What does it mean for my baby if the ROP is considered mild? And what if it’s severe?	5	4	5
6. Can environmental factors or care practices in the NICU influence the risk or severity of ROP in my baby?	5	3	4
7. Can ROP improve or worsen over time, and how quickly?	5	4	4
8. How is the progression of the disease monitored?	5	5	4
9. What are the chances of ROP resolving on its own without treatment?	5	4	4
10. Are there any lifestyle or environmental changes I can make to support my baby diagnosed with ROP?	5	5	5
**Treatment of Retinopathy of Prematurity**			
Does having ROP always mean my child will need treatment?	5	4	4
2. What options do we have for treating ROP?	5	5	2
3. Can you tell me the pros and cons of using laser therapy for ROP?	5	4	4
4. What should I know about the benefits and risks of intravitreal injections for ROP?	5	4	3
5. Under what conditions might my baby need surgery for ROP?	5	3	2
6. In cases where ROP is diagnosed, how critical is the timing between diagnosis and the initiation of treatment?	5	4	4
7. What symptoms should prompt me to bring my child in for an earlier ROP check-up after treatment?	5	4	4
8. Will my child require multiple treatment sessions for their ROP?	5	4	2
9. What are the possible side effects of treatments for ROP?	4	4	4
10. Are there any new treatments or research for ROP I should be aware of?	5	4	5
**Prognosis of Retinopathy of Prematurity**			
What can happen if ROP is not treated?	5	5	4
2. How likely is it that my child will become blind from ROP?	5	4	4
3. What long-term issues can ROP cause for my child?	5	4	5
4. What are the long-term effects of treating ROP with laser photocoagulation?	5	4	5
5. What long-term effects can come from treating ROP with intravitreal injections?	5	2	3
6. Should my child have regular eye check-ups even if they don’t have ROP after finishing their screenings?	5	3	4
7. If my premature baby didn’t develop ROP, is there still a risk of vision problems?	5	2	5
8. How can ROP affect my child’s life as they grow older?	5	4	5
9. Are there any activities or situations my child should avoid because of ROP?	5	4	5
10. What support and resources are available for families dealing with an ROP in long term?	5	5	5

A Likert scale from 1 to 5 was used, where 1 indicates ‘Strongly Disagree’ and 5 indicates ‘Strongly Agree’.

**Table 2:** Mean and median scores of three large language models across different question categories.

Category	ChatGPT Mean ± SD Median (Min–Max)	Bing Mean ± SD Median (Min–Max)	Gemini Mean ± SD Median (Min–Max)	*p* *
**General Information about Retinopathy of Prematurity (ROP)**	4.70 ± 0.94 5.00 (2–5)	4.60 ± 0.96 5.00 (2–5)	4.70 ± 0.67 5.00 (3–5)	0.735
**Screening of Retinopathy of Prematurity**	4.70 ± 0.48 5.00 (4–5)	3.40 ± 0.84 4.00 (2–4)	2.80 ± 1.03 2.50 (2–5)	**0.001**
**Diagnosis of Retinopathy of Prematurity**	5.00 ± 0.0 5.00 (5–5)	4.10 ± 0.99 4.00 (2–5)	4.10 ± 0.87 4.00 (2–5)	**0.009**
**Treatment of Retinopathy of Prematurity**	4.90 ± 0.31 5.00 (4–5)	4.00 ± 0.47 4.00 (3–5)	3.40 ± 1.07 4.00 (2–5)	**0.001**
**Prognosis of Retinopathy of Prematurity**	5.00 ± 0.0 5.00 (5–5)	3.70 ± 1.05 4.00 (2–5)	4.50 ± 0.70 5.00 (3–5)	**0.003**
**Total**	4.86 ± 0.49 5.00 (2–5)	3.96 ± 0.94 4.00 (2–5)	3.90 ± 1.11 4.00 (2–5)	**<0.001**

* Friedman test.

**Table 3:** Reliability and readability scores of three large language models.

Large Language Model	Reliability		Readability		
	DISCERN	EQIP	Flesch Reading Ease Score	Coleman-Liau Index	Flesch–Kincaid Grade Level
**ChatGPT**	63	72.2	28.7	15.27	13.5
**BingAI**	53	61.1	20.8	13.94	11.0
**Gemini**	57	63.8	39.1	14.53	11.6

For DISCERN, the scores are classified in five groups as excellent (i.e., 63–75 points), good (i.e., 51–62 points), fair (i.e., 39–50 points), poor (i.e., 27–38 points) or very poor (i.e., 16–26 points).

## Data Availability

The data presented in this study are available on request from the corresponding author (C.D.E.) due to privacy.
